# Future Challenge: Assessing the Antibiotic Susceptibility Patterns of *Staphylococcus* Species Isolated from Canine Otitis Externa Cases in Western Romania

**DOI:** 10.3390/antibiotics13121162

**Published:** 2024-12-02

**Authors:** János Dégi, Sorin Morariu, Florin Simiz, Viorel Herman, Florin Beteg, Diana Maria Dégi

**Affiliations:** 1 Department of Infectious Diseases and Preventive Medicine, University of Life Sciences “King Mihai I” from Timișoara, 300645 Timisoara, Romania; viorelherman@usvt.ro; 2 Department of Parasitology, Parasitic Diseases and Dermatology, University of Life Sciences “King Mihai I” from Timișoara, 300645 Timisoara, Romania; sorinmorariu@usvt.ro; 3 Department of Internal Medicine, University of Life Sciences “King Mihai I” from Timișoara, 300645 Timisoara, Romania; 4 Department of Clinical Sciences, University of Agricultural Sciences and Veterinary Medicine, Cluj Napoca, 400374 Cluj Napoca, Romania; florin.beteg@usamvcluj.ro; 5 Department of Toxicology and Toxicoses, Plant Biology and Medicinal Plants, University of Life Sciences “King Mihai I” from Timișoara, 300645 Timisoara, Romania; diana.maria-degi.fbira@usvt.ro

**Keywords:** antimicrobial resistance, otitis externa, dog, *Staphylococcus*

## Abstract

Introduction: Antimicrobial resistance (AMR) has surfaced as a critical challenge to public health on a global scale. The precise and swift identification of resistance to antimicrobial agents, along with timely and suitable antimicrobial therapy paired with effective stewardship practices, is crucial for managing the rise and dissemination of antimicrobial resistance. The objective of our investigation was to outline the antimicrobial resistance profile of *Staphylococcus* spp., a significant contributor to canine otitis, a prevalent condition in dogs, isolated in Western Romania. Methods and Materials: All data were collected from clinical cases of canine otitis externa which presented at the University Clinic of the Faculty of Veterinary Medicine in Timișoara/Romania. A clinical evaluation was conducted, from which era swabs are usually collected and sent for analysis at the laboratory. Laboratory analysis included the microbiological examination for identifying *Staphylococcus* spp. and determining antibiotic susceptibility phenotypes. Statistical analysis was implemented on all data that were collected. The ear swabs were processed with standard procedures for cultivating and identifying bacteria. The resulting subcultures were processed to determine the staphylococcal species on the GP ID Cards of the Vitek^®^ 2 automatic system. The antimicrobial susceptibility profiles were detected by the Vitek^®^ 2 system using an AST-GP80 card. These isolated *Staphylococcus* spp. strains were further processed by real-time PCR and PCR-RFLP. Results: Of all the auricular exudate samples analyzed, 76 were positive for *Staphylococcus* spp. (59.38%). Within these, in 82% of auricular samples, six distinct *Staphylococcus* spp. were identified (*Staphylococcus (S.) pseudintermedius, S. intermedius*, *S. hyicus*, *S. delphiny*, *S. shleiferi,* and *S. aureus*). Our data indicate that the PCR-RFLP assay is a practical approach to *S. pseudintermedius* identification, allowing for discrimination from the other *Staphylococcus Intermedius Group* (SIG) species and important staphylococcal pathogens of dogs. The highest frequency of resistant *S. pseudintermedius* isolates was detected against tetracycline (21/34; 61.76%; *p*-value 0.003), gentamicin (20/34; 58.82%), and kanamycin (20/34; 58.82%). Conclusions: These results are essential to guide the prudent use of antibiotics in veterinary medicine. They will also help design efficient control strategies and measure their effectiveness.

## 1. Introduction

Otitis externa (OE) is prevalent in dogs presenting to veterinary clinics worldwide [1,2,3]. OE is characterized by inflammation of the external ear canal, which may involve the entire length or any segment from the tympanic membrane (TM) to the outer meatus and is frequently accompanied by concomitant pineal alterations. The condition may be unpleasant and/or itchy, acute or chronic, and can impact one or both ears. Tissue alterations, including edema and glandular hyperplasia, may arise from the underlying etiology or subsequent infection, exacerbating the condition [4].

OE is a common pathology affecting canine ears globally, with prevalence rates between 5% and 20% [2,5,6]. This disorder is typically characterized as a multifactorial disease, primarily linked to damage or alterations in the ear environment due to various factors, including foreign bodies, endocrinopathies, autoimmune diseases, parasites, or allergies. Along with predisposing elements such as breed and ear conformation, these factors promote colonization by opportunistic pathogens [5].

The microbiota in a healthy canine ear is maintained through a sophisticated interplay of mechanical, chemical, immunological, and microbial interactions akin to those in the human ear [7,8,9]. Disruption of the microbiota can result in prolonged dysbiosis and the potential for secondary infections to occur. Consequently, numerous studies have concentrated on elucidating the function of the commensal microbiota in dogs’ ears concerning the development of otitis externa [10,11]. Identification through culture-based methods and, more recently, next-generation sequencing of the microbiome has revealed various Gram-positive and Gram-negative bacterial species associated with otitis externa. These include *Staphylococcus* spp., *Pseudomonas* spp., *Proteus* spp., *Escherichia coli*, and *Corynebacterium* spp. [11,12,13]. Additionally, opportunistic commensal yeasts such as *Malassezia pachydermatis* and *Candida albicans* have been identified, typically residing in the ear epithelium without causing harm under normal conditions [10,14].

Staphylococci are commensals and opportunistic pathogens in companion animals, responsible for various infections such as skin disorders, otitis, meningitis, and other infections [15,16,17]. The common species colonizing and infecting companion animals include *S. pseudintermedius*, *S. aureus*, *S. xylosus*, *S. epidermidis*, *S. delphini*, and *S. hyicus* [18,19].

Inadequate treatment protocols for canine otitis externa and the potential misuse of antimicrobial therapies may lead to the development of multidrug-resistant (MDR) microorganisms. They pose a considerable risk to global public health and are a primary focus of One Health projects [20,21,22]. The advancement of antimicrobial resistance in bacteria and the emergence of multidrug-resistant microorganisms pose considerable challenges for animal welfare and public health. Dogs carrying MDR pathogens tend to undergo several unsuccessful treatment attempts. The concern regarding public health, or the interconnectedness of human, animal, and environmental health, related to antimicrobial resistance and multidrug-resistant pathogens is rooted in the zoonotic potential of several prevalent bacteria found in cases of canine otitis externa. *S. pseudintermedius* and *S. aureus* are recognized as the predominant zoonotic microorganisms facilitating transmission between humans and pets [23]. *S. aureus* is typically associated with transmission from humans to companion animals, whereas *S. pseudintermedius* is more commonly identified in pet-to-human zoonoses [24,25,26]. *S. pseudintermedius* is an opportunistic pathogen causing various infections that are difficult to treat, mainly because of the development of antimicrobial resistance [27]. As in many bacteria, antibiotic resistance is an increasing problem in *Staphylococcus* infections, leading to significant challenges in combating disease in human and veterinary medicine. Methicillin-resistant *S. pseudintermedius* (MRSP) and multidrug-resistant *S. pseudintermedius*, including methicillin-susceptible and resistant variants (MDR-MSSP and MDR-MRSP), are found worldwide. MRSP is of particular concern because beta-lactam antibiotics are often the first treatment choice for *Staphylococcus*-associated infections.

Romania ranks among the European nations with the highest prevalence of multidrug-resistant (MDR) pathogens. In 2020, the peak resistance rates for fluoroquinolones, carbapenems, and aminoglycosides were witnessed [28,29]. Over the past few decades, the number of companion animals in developed nations has grown significantly. Cross-transmission of AMR characteristics and an increased risk of infection result from increased animal–human interaction. Antibiotic abuse or misuse alone is insufficient for such a massive transmission of resistant microorganisms between humans and pets, and several factors (population characteristics, geographic location, and investigative techniques) should be considered when defining the epidemiological scale of AMR transmission between humans and animals [30].

Therefore, this study aims to explore the antibiotic susceptibility of *Staphylococcus* spp. isolated from otitis externa in dogs to shed light on its potential impact on human and animal health. As a subsidiary objective, this investigation sought to identify the prevalent *Staphylococcus* species colonizing the external ear at the species level, thereby enhancing our understanding of which *Staphylococcus* spp. pose a heightened risk of inducing pathologies in their hosts.

## 2. Results

A total of 128 submissions were processed for diagnosis of canine external otitis at the bacterial diseases diagnostic laboratory of the Infectious Diseases and Preventive Medicine Department, Faculty of Veterinary Medicine Timisoara from 2020 to 2024 (2020, n = 27; 2021, n = 30; 2022, n = 21; 2023, n = 23; 2024, n = 27). The age of the dogs ranged from 4 months to 15 years, with a median of 8 years (IQR: 5–11). Regarding age groups, 1.3% of the animals were younger than 1 year, 27.5% from 1 to 10 years, and 66.4% older than 10. No statistically significant differences were observed between the age categories and culture results (*p*-value 0.323). In total, 84 animals were male (65.62%; 95% CI: 1.2713 to 1.8118%, *p*-value 0.001) and 44 female (34.38%; 95% CI: 2.427 to 3.4589%, *p*-value 0.0001). A total of 12 different dog breeds were described. Mixed breed dogs were the most frequently observed (n = 42; 32.81%; 95% CI: 2.5426 to 3.6236%), followed by the Labrador Retriever (n = 28; 21.87.9%; 95% CI: 3.8138 to 5.4354%), German Shepherd (n = 18; 14.06%; 95% CI: 5.9326 to 8.4551%), Cocker Spaniel (n = 14; 10.93%; 95% CI: 7.6277 to 10.8709%), French Bulldog (n = 10; 7.81%; 95% CI: 10.679 to 15.219%), and Yorkshire Terrier (n = 6; 4.68%; 95% CI: 17.798 to 25.365%). Cocker Spaniel was the only breed significantly associated with a higher rate of *Staphylococcus* spp.-positive ear culture results (11/76; 14.47%, *p*-value 0.001).

### 2.1. Bacteriological Results

Of all the auricular exudate samples analyzed, 76 were positive for *Staphylococcus* spp. (59.38%, 95% CI: 0.4678 to 0.7432; *p*-value < 0.003) and were evaluated for their antimicrobial profiles. Within these, 82% were positive for colonies compatible with *Staphylococcus* species, indicating the presence of at least two or more species of the genus *Staphylococcus* in these isolates. Six distinct *Staphylococcus* spp. were identified. Within the bacterial species examined, *S. pseudintermedius* was the most prevalent microorganism detected (n = 34; 44.73%; 95% CI: 0.3098 to 0.6252; *p*-value < 0.001), followed by *S. intermedius* (n = 14; 18.42%; 95% CI: 0.3098 to 0.6252; *p*-value < 0.003), *S. hyicus* (n = 8; 10.52%; 95% CI: 0.0454 to 0.2074; *p*-value < 0.001), *S. delphini* (n = 7; 9.21%; 95% CI: 0.03703 to 0.18977; *p*-value < 0.001), *S. schleiferi* (n = 7; 9.21; 95%; 95% CI: 0.03703 to 0.18977; *p*-value < 0.001), and *S. aureus* (n = 6; 7.89%; 95% CI: 0.02897 to 0.17184; *p*-value < 0.001). Table 1 presents the distribution of the detected *Staphylococcus* spp. according to the Vitek 2 GP ID card.

The Kolmogorov–Smirnov test results indicated a nonsignificant difference from the normal distribution (D (6) = 0.33, *p* = 0.426, and Sample Standard deviation = 10.838). These samples were further processed by real-time PCR.

### 2.2. Results of PCR

The *nuc* gene is found in every isolate of *S. aureus* and has also been detected in other *Staphylococcus* species, notably in *S. pseudintermedius*, *S. intermedius*, *S. hyicus*, *S. schleiferi*, and *S. delphini*. Of the 76 isolates, 53 (69.73%) possess the *nuc* gene. The application of the selected primers resulted in the identification of this gene across six distinct *Staphylococcus* species: *S. aureus* (6/6; 100%), *S. pseudintermedius* (30/34; 88.23%), *S. intermedius* (8/14; 57.14%), *S. delphini* (1/7; 14.28%), *S. hyicus* (4/8; 50%), and *S. schleiferi* (4/7; 57.14%).

PCR-RFLP genotyping successfully amplified a 320 bp *pta* gene from all examined strains. The PCR products of *S. pseudintermedius* exhibited a single *MboI* site, yielding two restriction fragments measuring 215 bp and 109 bp, respectively. In contrast, the SIG species *S. delphini*, *S. intermedius*, and the *S. schleiferi* strains lacked an *MboI* restriction site. *S. aureus* isolates exhibited a distinct *MboI* site, producing restriction fragments of 158 bp and 167 bp that manifested as a single band following agarose electrophoresis; this band was easily differentiated from the restriction fragment profile of *S. pseudintermedius*.

### 2.3. Results of Antimicrobial Susceptibility Test

Of the 76 isolates tested, all were due to acceptable growth in the control well of the Vitek 2 automated susceptibility testing (AST) card. The obtained results were automatically correlated with the standards for antimicrobial susceptibility test of the Clinical and Laboratory Standards Institute (CLSI), M100-S24 [31].

The antimicrobial susceptibility profiles of the identified *Staphylococcus* species, as determined by the Vitek^®^ 2 system, are presented in Table 2. The highest occurrence of resistant *S. pseudintermedius* isolates was identified against tetracycline (21/34; 61.76%; *p*-value 0.0796), gentamicin (20/34; 58.82%), and kanamycin (20/34; 58.82%). For *S. intermedius*, all isolates were susceptible to marbofloxacin and pradofloxacin. The highest susceptibility rates were found for cefovecin (13/14; 92.85%; *p*-value < 0.847) and enrofloxacin (12/14; 85.71%). Nitrofurantoin (5/7; 71.42%; *p*-value < 0.563) and clindamycin (5/7; 71.42%; *p*-value < 0.563) presented the highest susceptibility rates for *S. schleiferi*, while doxycycline (3/7; 42.85%; *p*-value < 0.205) and gentamycin (2/7; 28.57%) were the antimicrobials with the lowest susceptibility rates for this pathogen. For *S. hyicus*, cefovecin, oxacillin, marbofloxacin, pradofloxacin (8/8; 100%), and clindamycin (7/8; 87.5%) showed the highest susceptibility rates. *S. aureus* was only represented by one isolate with 100% susceptibility rates to pradofloxacin. Tetracyclin (5/7; 71.42%: *p*-value 0.563) and penicillin (5/7; 71.42.1; *p*-value < 0.563) displayed the highest rates of resistant phenotypes to *S. delphini*. Our findings indicate that 82.8% (63/76) of the isolates exhibited MDR phenotypes.

## 3. Discussion

Many microorganisms are involved in the pathogenesis of this condition; therefore, a good knowledge of pathogen distribution and updated antimicrobial susceptibility data are essential for efficiently handling the condition [32,33].

One critical problem in clinical settings requiring confirmatory phenotyping of the staphylococci causing OE in dogs is the inability to differentiate between *S. aureus* and *S. pseudintermedius* because of the lack of differentiation tests between these two species [30], which can result in incorrect diagnoses. Diagnostic laboratories in our clinical settings are more accustomed to phenotyping *S. aureus* isolates. However, *S. pseudintermedius* is the most frequent agent causing otitis externa in dogs; they report *S. aureus* [34]. The data suggest that the PCR-RFLP assay effectively identifies *S. pseudintermedius*, enabling differentiation from other SIG species and significant staphylococcal pathogens in dogs.

Molecular surveillance of *Staphylococcus* spp. indicates a necessity for swift and reliable techniques for detecting and identifying organisms significant to clinical and epidemiological contexts. The *nuc* gene sequence, which encodes thermonuclease, facilitates the development of specific primers for the molecular identification of *S. pseudintermedius* through PCR. Primers were specifically designed to amplify a segment of the *nuc* gene, which encodes the thermostable endonuclease of coagulase-positive *Staphylococcus*. Given the discrepancy concerning the etiology of OE in our clinical setting, we hypothesized that *S. pseudintermedius*, rather than the frequently diagnosed *S. aureus*, was the staphylococcal species responsible for OE in our patients.

Precise, traditional methods employed in clinical microbiology for profiling antimicrobial susceptibility tend to be labor-intensive and somewhat costly. Consequently, medical professionals frequently recommend empirical antimicrobial treatments and broad-spectrum antibiotics. While newly developed AST systems demonstrate benefits over conventional approaches in terms of testing speed and the ability to offer a more profound understanding of resistance mechanisms, thorough validation is essential in order to implement these techniques in clinical settings. The factors contributing to antimicrobial resistance are intricate and varied. The use of antibiotics in patients suspected of having moderate to severe bacterial infections is inappropriate in at least 50% of instances [35]. Antimicrobial stewardship (AMS) represents a crucial approach in the fight against resistance.

Staphylococci are prevalent constituents of the skin microbiota in dogs, particularly in moist regions, and frequently act as opportunistic pathogens in otitis externa and various skin infections. The isolation rates of *S. pseudintermedius* in cases of canine otitis externa exhibit variability. The findings align with OʹNeill et al. [5], who reported a 7.30% prevalence in a sample of 22,333 dogs diagnosed with otitis. In contrast, other studies have reported significantly higher isolation frequencies for *S. pseudintermedius*, ranging from 39.2% to 58.8% [1,20,31].

*S. schleiferi* (2.9%) and *S. aureus* (1.8%) have frequently been isolated within the *Staphylococcus* genus. *S. schleiferi* is an emerging zoonotic pathogen in humans and animals, gaining significance in canine ear and skin infections. *S. aureus* is implicated in numerous animal and human pathologies [36]. The occurrence of both pathogens in canine otitis externa is typically low, with rates between 4.9% and 6.2% [19,20]. Due to their zoonotic potential and capacity to harbor various resistance mechanisms, the significance of these two microorganisms in canine otitis externa and public health warrants attention [37].

*S. pseudintermedius* demonstrates the third greatest prevalence of antimicrobial-resistant isolates (25.1%). Penicillin G (69%), tetracycline (41.7%), and kanamycin (30.7%) exhibit the highest frequencies of resistance phenotypes. Resistance to Penicillin G, as is resistance to tetracycline, is frequently observed in canine otitis externa cases [19,38]. Oxacillin (methicillin) resistance in *S. pseudintermedius* derived from canine otitis cases has been documented globally, with prevalence rates between 8.9% and 50% [19,39,40]. The appearance of this phenotype is critically essential to animal health, given that numerous MRSP isolates are associated with multidrug resistance [40]. Our findings indicate that 82.8% of the isolates exhibited MDR phenotypes, underscoring the necessity of comprehending the epidemiology of this pathogen to develop effective control strategies. Furthermore, findings from a recent investigation conducted in Brazil validated the efficient transmission of MRSP between dogs and their owners [37,41], highlighting the zoonotic risk associated with this pathogen. In Spain, MRSPs have been identified in healthy dogs, showing a prevalence of 4.6%. A study conducted in Spain reported a prevalence of over 20% for oxacillin-resistant *Staphylococcus* spp. in clinical samples, encompassing wound infections, dermatitis, otitis, abscesses, conjunctivitis, and respiratory tract infections [42]. However, the specific percentage of MRSP in otitis cases was not provided. The study identified methicillin-resistant staphylococci, which comprised one *S. schleiferi* (6.25%), three *S. aureus* (30%), and six *S. lentus* (100%). This highlights the significance of *Staphylococcus spp*. as a reservoir for methicillin-resistant bacterial phenotypes, posing potential risks to both animal and public health [43,44,45].

Tesin et al. [11], in a study conducted on 60 dogs with otitis externa, described the fact that *S. pseudintermedius* was the most resistant to penicillin (76%) and amoxicillin (69%). In addition, high resistance to clindamycin (66%), lincomycin, and azithromycin, represented by 62%, has also been shown. On the contrary, these bacteria have shown low resistance against cefoxitin (3%), amoxicillin + clavulanic acid (10%), ceftriaxone (14%), and enrofloxacin (14%).

## 4. Materials and Methods

All data were collected from clinical cases of canine otitis externa which presented at the University Clinic of the Faculty of Veterinary Medicine in Timișoara/Romania for a clinical evaluation, from which era swabs are usually collected and sent for analysis at the laboratory. The cases were from 2020 to 2024 and were registered in our clinic IT system (AtlasVet Clinic, Romania). The dogs in the study were monitored for breed, sex, age (they were categorized: <1 year, 1 to 10 years, >10 years), and medical history. The auricular samples were collected from all dogs with clinical symptoms of otitis externa.

The ear swabs were transported to the Bacteriology Laboratory of the Infectious Diseases Department at the Faculty of Veterinary Medicine in Timisoara, which included standard procedures for cultivating and identifying bacteria. Columbia agar supplemented with 5% ram blood and Chapman and MacConkey agar (bioMérieux, Marcy l’Etoile, France) were used. The resulting subcultures were processed to determine the staphylococcal species on the GP ID Cards of the Vitek^®^ 2 automatic system (identification panel, GP ID card; software, Vitek^®^ 2 Systems version 07.01; bioMérieux, Marcy l’Etoile, France) using the manufacturer’s default instructions. Antibiotic susceptibility testing was conducted by utilizing Vitek^®^ 2 preset antimicrobial card AST-GP80 to characterize Gram-positive bacteria (bioMérieux, Marcy l’Etoile, France) with Vitek^®^ 2 Advanced Expert System software (version 8.01) [46,47].

A descriptive analysis of antimicrobial multidrug-resistant phenotypes was conducted on the predominant *Staphylococcus* pathogens identified, namely *S. pseudintermedius*, *S. aureus*, *S. schleiferi*, *S. intermedius*, *S. delphini*, and *S. hyicus*. MDR denotes acquired resistance to at least one antimicrobial agent across three or more antimicrobial classes.

All acquired data were statistically analyzed.

### 4.1. DNA Extraction and PCR Protocol for Staphylococcal Species

The bacterial isolates were grown in brain heart infusion (BHI) broth (MLT, Arad, Romania) with the addition of 0.6% yeast extract (Roth, Bucharest, Romania), then incubated at 37 °C for 24 h. Then, aliquots of broth cultures were used for the DNA extraction. A sample was collected from each isolate compatible with *Staphylococcus* species, and the genetic material was extracted according to the protocol described by González-Domínguez et al. [34] and Bannoehr et al. [48]. DNA quality was evaluated by determining the concentration through spectrophotometry using a Vis DLAB SP-V1000 spectrophotometer (DLAB, Senai, Malaysia); its purity was analyzed by the relationship between absorbance at 260 and 280 nm.

Specific primers were designed to amplify a *nuc* gene segment by real-time PCR. The gene sequence for each *Staphylococcus* species considered in this study was aligned using the GenBank/NCBI software (version 3.0) to identify sequence homologies shared among the *nuc* genes [49,50]. 

Amplification was achieved using the commercial kit QuantiNova^®^ SYBR^®^ Green PCR Kit (Qiagen^®^, Germantown, MD, USA). The identity of all isolates was confirmed using a thermonuclease (*nuc*) gene PCR test that can differentiate between *S. aureus*, *S. delphini* (groups A and B), *Staphylococcus hyicus*, *S. intermedius*, *S. pseudintermedius*, and *Staphylococcus schleiferi*. The *nuc* PCR test results were accepted as the reference method for species-level identification. Accurate identification of *S. pseudintermedius* has important implications for interpreting antimicrobial susceptibility testing data and may be necessary for other group members. PCR conditions were optimized using positive control strains ATCC 25923 (*S*. *aureus*), ATCC 29663 (*S*. *intermedius*), ATCC 51874 (*S. pseudintermedius*), ATCC 49172 (*S*. *delphyni*), ATCC 43808 (*S*. *schleiferi*), and ATCC l1249 (*S*. *hyicus*).

For the identification of *S. pseudintermedius* and its discrimination from other SIG species and several other important staphylococcal pathogens, a simple and effective method is PCR RFLP, based on sequence analysis of one of the loci, namely the *pta* gene, which encodes the enzyme phosphoacetyltransferase, highlighting the presence of a *MboI* restriction site [51]. The oligonucleotide primers used for the molecular investigations were the following: pta_f1, AAA GAC AAA CTT TCA GGT AA, pta_r1, GCA TAA ACA AGC ATT GTA CCG. The DNA extractions and PCR methods were carried out at the Molecular Biology Laboratory, CLCHC Research Laboratory Complex, King Mihai I University of Life Sciences from Timisoara.

### 4.2. Statistical Analysis

The normality of the distribution of the variables was checked using the Kolmogorov–Smirnov statistical test.

Continuous variables exhibiting normal distribution were provided as 95% confidence intervals (CIs); non-normal variables were reported as medians with interquartile ranges (IQRs). The association between variables was analyzed using a one-way Chi-square test. Statistical analysis was performed using IBMR SPSSR Statistic version 25 (IBM Corp., Armonk, NY, USA). A *p*-value < 0.05 was considered significant.

## 5. Conclusions

Considering the zoonotic potential of *Staphylococcus* spp., it is crucial to emphasize the necessity of an augmented focus on research and academic study within the field of canine dermatology. This is particularly pertinent for investigations centered around *Staphylococcus* species. Molecular surveillance of *Staphylococcus* spp. indicates a necessity for swift and reliable techniques for detecting and identifying organisms significant to clinical and epidemiological contexts. Antibiotics are among human and veterinary medicine’s most essential treatment options, but AMR is a serious public health concern. Our findings indicate that 82.8% of the isolates exhibited MDR phenotypes, underscoring the necessity of comprehending the epidemiology of this pathogen to develop effective control strategies. Our study focuses on reducing antibiotic use and encompasses infection control, clinical microbiology, the surveillance of antibiotic use and AMR, pharmacovigilance, education, guidelines, and regulations. However, as our study highlights, a shared commitment between the animal and human medicine worlds has added value in the fight against AMR. It represents a practical application of the One Health approach.

## Figures and Tables

**Table 1:** Distribution frequency of *Staphylococcus* spp. as determined by Vitek^®^ 2 system identification (Vitek 2^®^ GP ID Card).

Bacterial Species	No. of Isolated Species (n = 76)	% (95% CI)	*p*-Value/Chi-Squared
*S. pseudintermedius*	34	44.73 (0.3098 to 0.6252)	0.001/16.0364
*S. intermedius*	14	18.42 (0.1007 to 0.3091)	0.003/8.3333
*S. hyicus*	8	10.52 (0.0454 to 0.2074)	0.001/16.0952
*S. delphini*	7	9.21 (0.03703 to 0.18977)	0.001/17.780
*S. schleiferi*	7	9.21 (0.03703 to 0.18977)	0.001/17.780
*S. aureus*	6	7.89 (0.02897 to 0.17184)	0.001/19.600

**Table 2:** Details of the antimicrobial susceptibility test results of *Staphylococcus* spp. identified from dogs’ otitis externa, as detected by Vitek^®^ 2 (AST-GP80, Ref 421826, BioMérieux, Paris, France). The numbers represent the percentages (%) of resistance, sensitivity, intermediate sensitivity, and number of strains (n).

Antibiotics (Drugs Family)	*S. intermedius* (n = 14)		*S. aureus* (n = 6)		*S. hyicus* (n = 8)		*S. delphini* (n = 7)		*S. scleiferi* (n = 7)		*S. aureus* (n = 6)	
	S	R	S	R	S	R	S	R	S	R	S	R
AMC (BL)	82.35 (28)	17.64 (6)	78.58 (11)	14.28 (2)	62.5 (5)	12.5 (1)	85.71 (6)	14.28 (1)	71.42 (5)	28.57 (2)	33.33 (2)	50.0 (3)
P (BL)	35.29 (12)	52.94 (18)	27.27 (3)	64.28 (9)	37.5 (3)	50.0 (4)	28.57 (2)	71.42 (5)	57.14(4)	42.85 (3)	(0)	83.33 (5)
CF (BL)	94.11 (32)	5.88 (2)	71.42 (10)	28.57 (4)	75 (6)	12.5 (1)	71.42 (5)	14.28 (1)	85.71 (6)	(0)	66.667 (4)	33.33 (2)
CFO (BL)	100 (34)	(0)	92.85 (13)	(0)	100 (8)	(0)	100 (7)	(0)	100 (7)	(0)	83.33 (5)	16.67 (1)
OX1 (BL)	97.05 (33)	2.94 (1)	78.58 (11)	14.28 (2)	100 (8)	(0)	100 (7)	(0)	100 (7)	(0)	33.33 (2)	66.67 (4)
OXSF	Negative		Negative		Negative		Negative		Negative		Positive	
GM (AM)	32.35 (11)	58.82 (20)	27.27 (3)	71.42 (10)	25.0 (2)	50.0 (4)	42.85 (3)	42.85 (3)	28.57 (2)	57.14 (4)	(0)	83.33 (5)
K (AM)	27.46 (9)	58.82 (20)	35.71 (5)	64.28 (9)	37.5 (3)	37.5 (3)	57.14 (4)	42.85 (3)	71.42 (5)	28.57 (2)	16.66 (1)	83.33 (5)
N (AM)	38.23 (13)	55.88 (19)	50.0 (7)	50.0 (7)	75.0 (6)	25.0 (2)	71.42 (5)	14.28 (1)	57.14 (4)	42.85 (3)	33.33 (2)	66.67 (4)
MRB (FQ)	100 (34)	(0)	100 (14)	0	100 (8)	(0)	100 (7)	(0)	100 (7)	(0)	83.33 (5)	16.67 (1)
ENR (FQ)	94.11 (32)	5.88 (2)	85.71 (12)	14.28 (2)	75.0 (6)	12.5 (1)	100 (7)	(0)	100 (7)	(0)	66.667 (4)	33.33 (2)
PRA (FQ)	100 (34)	(0)	100 (14)	0	100 (8)	(0)	100 (7)	(0)	100 (7)	(0)	100 (6)	0
DO (TE)	32.35 (11)	58.82 (20)	28.57 (4)	57.14 (8)	37.5 (3)	50.0 (4)	28.57 (2)	57.14 (4)	42.85 (3)	57.14 (4)	16.66 (1)	66.67 (4)
TE (TE)	26.47 (9)	61.76 (21)	21.42 (3)	71.42 (10)	37.5 (3)	62.5 (5)	28.57 (2)	71.42 (5)	42.85 (3)	42.85 (3)	16.66 (1)	83.33 (5)
E (MA)	52.94 (18)	41.17 (14)	64.28 (9)	28.57 (4)	50.0 (4)	50.0 (4)	71.42 (5)	14.28 (1)	57.14 (4)	28.57 (2)	16.66 (1)	66.67 (4)
ICR	Negative		Negative		Negative		Negative		Negative		Positive	
C (Ph)	64.70 (22)	26.47 (9)	78.57 (11)	14.28 (2)	62.5 (5)	25.0 (2)	57.14 (4)	28.57 (2)	57.14 (4)	42.85 (3)	50.0 (3)	33.33 (2)
CM (Li)	85.29 (29)	14.70 (5)	26.47 (9)	28.57 (4)	87.5 (7)	(0)	85.71 (6)	14.28 (1)	71.42 (5)	(0)	33.33 (2)	50.0 (3)
FT (NIT)	38.23 (13)	55.88 (19)	64.28 (9)	28.57 (4)	50.0 (4)	50.0 (4)	57.14 (4)	28.57 (2)	71.42 (5)	28.57 (2)	33.33 (2)	66.67 (4)
SXT (TS)	52.94 (18)	35.29 (12)	57.14 (8)	35.71 (5)	50.0 (4)	75.0 (3)	42.85 (3)	57.14 (4)	71.42 (5)	28.57 (2)	16.66 (1)	83.33 (5)

The expression is a percentage and exact number, representing the number of strains. Antimicrobial abbreviations: AMC = Amoxicillin/clavulanate; P = Penicillin G; CF = Cephalothin; CFO = Cefovecin; OX1—oxacillin; OXSF—Cefoxitin screen test; GM—Gentamicin; K = Kanamycin; N = Neomycin; MRB = Marbofloxacin; ENR = Enrofloxacin; PRA—Pradofloxacin; DO—Doxycycline; TE—Tetracycline; E—Erythromycin; ICR—Inducible clindamycin resistance; C—Chloramphenicol; CM—Clindamycin; FT—Nitrofurantoin; SXT = Sulfamethoxazole-trimethoprim. S: Susceptible; R: Resistant. Drugs Family abbreviations: BL—β-Lactams; AM—Aminoglycosides; FQ—Fluoroquinolones; TE—Tetracyclines; MA—Macrolide; Ph—Phenicols; Li—Lincosamides; NIT—Nitrofurans; TS—folate pathway inhibitors. When the antimicrobial susceptibility patterns were analyzed by antimicrobial class, aminoglycosides presented the highest rate of AMR (53.06% of the isolates) and folate pathway inhibitors (52.50% of the isolates). The fluoroquinolones class displayed the highest susceptibility rate (94.71%), followed by β-Lactams (73.30%) (Table 3).

**Table 3:** Distribution of antimicrobial susceptibility phenotypes per antimicrobial class among the *Staphylococcus* spp. using the Vitek^®^ 2 system (the number represents the average of individual antibiotics).

Antimicrobial Class	*S. pseudintermedius*	*S. intermedius*	*S. hyicus*	*S. delphini*	*S. schleiferi*	*S. aureus*	Mean
	R (%)	R (%)	R (%)	R (%)	R (%)	R (%)	R (%)
β-Lactams	15.88	24.82	15	20.0	14,284	50	23.33
Aminoglycosides	57.84	61.9	37.5	33.32	42.85	77.76	53.06
Fluoroquinolones	5.88	14.28	12.5	0	0	16.66	18.10
Tetracyclines	40,19	42.85	37.5	42.85	33.33	50.0	41.12
Macrolide	41.17	28.57	50.0	14.28	28.57	66.67	38.20
Phenicols	26.47	14.28	25.0	28.57	42.85	33.33	28.41
Lincosamides	14.70	28.57	0	14.28	0	50.0	17.92
Nitrofurans	55.88	28.57	50.0	28.57	28.57	66.67	43.03
Folate Pathway Inhibitors	35.29	35.71	75.0	57.14	28.57	83.33	52.50

R: resistant.

## Data Availability

The data supporting this study’s findings are available from the corresponding author upon reasonable request.

## References

[1] Dégi J., Imre K., Catana N., Morar A., Sala C., Herman V. (2013). Frequency of isolation and antibiotic resistance of staphylococcal flora from external otitis of dogs. Vet. Rec..

[2] Secker B., Shaw S., Atterbury R.J. (2023). *Pseudomonas* spp. in Canine Otitis Externa. Microorganisms.

[3] Ponn P.C., Tipold A., Volk A.V. (2024). Can We Minimize the Risk of Dogs Developing Canine Otitis Externa?—A Retrospective Study on 321 Dogs. Animals.

[4] Kaimio M., Malkamäki S., Kaukonen M., Ahonen S., Hytönen M.K., Rantala M., Lohi H., Saijonmaa-Koulumies L., Laitinen-Vapaavuori O. (2021). Clinical and Genetic Findings in 28 American Cocker Spaniels with Aural Ceruminous Gland Hyperplasia and Ectasia. J. Comp. Pathol..

[5] O’Neill D.G., Volk A.V., Soares T., Church D.B., Brodbelt D.C., Pegram C. (2021). Frequency and predisposing factors for canine otitis externa in the UK—A primary veterinary care epidemiological view. Canine Med. Genet..

[6] Terziev G., Borissov I. (2018). Prevalence of ear diseases in dogs—A retrospective 5-year clinical study. Bulg. J. Vet. Med..

[7] Ngo J., Taminiau B., Fall P.A., Daube G., Fontaine J. (2018). Ear canal microbiota—A comparison between healthy dogs and atopic dogs without clinical signs of otitis externa. Vet. Dermatol..

[8] Leonard C., Picavet P.P., Fontaine J., Clercx C., Taminiau B., Daube G., Claeys S. (2023). The Middle Ear Microbiota in Healthy Dogs Is Similar to That of the External Ear Canal. Vet. Sci..

[9] Moog F., Mivielle J., Brun J., Dumitrache M.O., Amalric N., Lecru L.-A., Pressanti C., Kondratjeva J., Combarros D., Fantini O. (2022). Clinical and Microbiological Performances and Effects on Lipid and Cytokine Production of a Ceruminolytic Ear Cleaner in Canine Erythemato-Ceruminous Otitis Externa. Vet. Sci..

[10] Borriello G., Paradiso R., Catozzi C., Brunetti R., Roccabianca P., Riccardi M.G., Cecere B., Lecchi C., Fusco G., Ceciliani F. (2020). Cerumen microbial community shifts between healthy and otitis affected dogs. PLoS ONE.

[11] Tesin N., Stojanovic D., Stancic I., Kladar N., Ružić Z., Spasojevic J., Tomanic D., Kovacevic Z. (2023). Prevalence of the microbiological causes of canine otitis externa and the antibiotic susceptibility of isolated bacterial strains. Pol. J. Vet. Sci.

[12] Tang S., Prem A., Tjokrosurjo J., Sary M., Van Bel M.A., Rodrigues-Hoffmann A., Kavanagh M., Wu G., Van Eden M.E., Krumbeck A. (2020). The canine skin and ear microbiome: A comprehensive survey of pathogens implicated in canine skin and ear infections using a novel next-generation-sequencing-based assay. Vet. Microbiol..

[13] Pye C. (2018). *Pseudomonas* otitis externa in dogs. Can. Vet. J..

[14] Harvey R.A. (2022). Review of Recent Developments in Veterinary Otology. Vet. Sci..

[15] János D., Viorel H., Ionica I., Corina P., Tiana F., Roxana D. (2021). Carriage of Multidrug Resistance Staphylococci in Shelter Dogs in Timisoara, Romania. Antibiotics.

[16] Haag A.F., Fitzgerald J.R., Penadés J.R. (2019). *Staphylococcus aureus* in Animals. Microbiol. Spectr..

[17] Gherardi G., Di Bonaventura G., Savini V., Savini V. (2018). Chapter 1—Staphylococcal Taxonomy. Pet-To-Man Travelling Staphylococci.

[18] Battaglia M., Garrett-Sinha L.A. (2023). *Staphylococcus xylosus* and *Staphylococcus aureus* as commensals and pathogens on murine skin. Lab. Anim. Res..

[19] Schwarz S., Feßler A.T., Loncaric I., Wu C., Kadlec K., Wang Y., Shen J. (2018). Antimicrobial resistance among staphylococci of animal origin. Microbiol. Spectr..

[20] Rosales R.S., Ramírez A.S., Moya-Gil E., de la Fuente S.N., Suárez-Pérez A., Poveda J.B. (2024). Microbiological Survey and Evaluation of Antimicrobial Susceptibility Patterns of Microorganisms Obtained from Suspect Cases of Canine Otitis Externa in Gran Canaria, Spain. Animals.

[21] Caneschi A., Bardhi A., Barbarossa A., Zaghini A. (2023). The Use of Antibiotics and Antimicrobial Resistance in Veterinary Medicine, a Complex Phenomenon: A Narrative Review. Antibiotics.

[22] Naziri Z., Poormaleknia M., Ghaedi Oliyaei A. (2022). Risk of sharing resistant bacteria and/or resistance elements between dogs and their owners. BMC Vet. Res..

[23] Roberts E., Nuttall T.J., Gkekas G., Mellanby R.J., Fitzgerald J.R., Paterson G.K. (2024). Not just in man’s best friend: A review of *Staphylococcus pseudintermedius* host range and human zoonosis. Res. Vet. Sci..

[24] Moses I.B., Santos F.F., Gales A.C. (2023). Human Colonization and Infection by *Staphylococcus pseudintermedius*: An Emerging and Underestimated Zoonotic Pathogen. Microorganisms.

[25] Morais C., Costa S.S., Leal M., Ramos B., Andrade M., Ferreira C., Abrantes P., Pomba C., Couto I. (2023). Genetic diversity and antimicrobial resistance profiles of *Staphylococcus pseudintermedius* associated with skin and soft-tissue infections in companion animals in Lisbon, Portugal. Front. Microbiol..

[26] Dégi J., Herman V., Igna V., Dégi D.M., Hulea A., Muselin F., Cristina R.T. (2022). Antibacterial Activity of Romanian Propolis against *Staphylococcusaureus* Isolated from Dogs with Superficial Pyoderma: In Vitro Test. Vet. Sci..

[27] Bajwa J. (2019). Canine otitis externa—Treatment and complications. Can. Vet. J..

[28] Mereuţă A.I., Docquier J.D., Rossolini G.M., Buiuc D. (2007). Detection of metallo-beta-lactamases in Gram-negative bacilli isolated in hospitals from Romania-research fellowship report. Bacteriol. Virusol. Parazitol. Epidemiol..

[29] Barbu I.C., Gheorghe-Barbu I., Grigore G.A., Vrancianu C.O., Chifiriuc M.C. (2023). Antimicrobial Resistance in Romania: Updates on Gram-Negative ESCAPE Pathogens in the Clinical, Veterinary, and Aquatic Sectors. Int. J. Mol. Sci..

[30] Rendle D.I., Page S.W. (2018). Antimicrobial resistance in companion animals. Equine Vet. J..

[31] Clinical and Laboratory Standards Institute (CLSI) (2014). Performance Standards for Antimicrobial Susceptibility Testing.

[32] Huang H.P., Little C.J., McNeil P.E. (2009). Histological changes in the external ear canal of dogs with otitis externa. Vet. Dermatol..

[33] Nuttall T. (2023). Managing recurrent otitis externa in dogs: What have we learned and what can we do better?. J. Am. Vet. Med. Assoc..

[34] González-Domínguez M.S., Carvajal H.D., Calle-Echeverri D.A., Chinchilla-Cárdenas D. (2020). Molecular Detection and Characterization of the mecA and nuc Genes From *Staphylococcus* Species (*S. aureus*, *S. pseudintermedius*, and *S. schleiferi*) Isolated From Dogs Suffering Superficial Pyoderma and Their Antimicrobial Resistance Profiles. Front. Vet. Sci.

[35] Nocera F.P., Pizzano F., Masullo A., Cortese L., De Martino L. (2023). Antimicrobial Resistant *Staphylococcus* Species Colonization in Dogs, Their Owners, and Veterinary Staff of the Veterinary Teaching Hospital of Naples, Italy. Pathogens.

[36] Gajic I., Kabic J., Kekic D., Jovicevic M., Milenkovic M., Mitic Culafic D., Trudic A., Ranin L., Opavski N. (2022). Antimicrobial Susceptibility Testing: A Comprehensive Review of Currently Used Methods. Antibiotics.

[37] Leonard C., Thiry D., Taminiau B., Daube G., Fontaine J. (2022). External Ear Canal Evaluation in Dogs with Chronic Suppurative Otitis Externa: Comparison of Direct Cytology, Bacterial Culture and 16S Amplicon Profiling. Vet. Sci..

[38] Guimarães L., Teixeira I.M., da Silva I.T., Antunes M., Pesset C., Fonseca C., Santos A.L., Côrtes M.F., Penna B. (2023). Epidemiologic Case Investigation on the Zoonotic Transmission of Methicillin-Resistant *Staphylococcus pseudintermedius* among Dogs and Their Owners. J. Infect. Public Health.

[39] Rana E.A., Nizami T.A., Islam M.S., Sarker S., Rahman H., Hoque A., Rahman M. (2024). Antimicrobial resistance and virulence profiling of *Staphylococcus pseudintermedius* isolated from cats, Bangladesh. Vet. Q..

[40] Suepaul S., Georges K., Unakal C., Boyen F., Sookhoo J., Ashraph K., Yusuf A., Butaye P. (2021). Determination of the frequency, species distribution and antimicrobial resistance of staphylococci isolated from dogs and their owners in Trinidad. PLoS ONE.

[41] Lord J., Millis N., Jones R.D., Johnson B., Kania S.A., Odoi A. (2022). Patterns of antimicrobial, multidrug and methicillin resistance among *Staphylococcus* spp. isolated from canine specimens submitted to a diagnostic laboratory in Tennessee, USA: A descriptive study. BMC Vet. Res..

[42] Gómez-Sanz E., Torres C., Lozano C., Sáenz Y., Zarazaga M. (2011). Detection and characterization of methicillin-resistant *Staphylococcus pseudintermedius* in healthy dogs in La Rioja, Spain. Comp. Immunol. Microbiol. Infect. Dis..

[43] Silva V., Araújo S., Monteiro A., Eira J., Pereira J.E., Maltez L., Igrejas G., Lemsaddek T.S., Poeta P. (2023). *Staphylococcus aureus* and MRSA in Livestock: Antimicrobial Resistance and Genetic Lineages. Microorganisms.

[44] Røken M., Iakhno S., Haaland A.H., Bjelland A.M., Wasteson Y. (2024). The Home Environment Is a Reservoir for Methicillin-Resistant Coagulase-Negative Staphylococci and Mammaliicocci. Antibiotics.

[45] Crespo-Piazuelo D., Lawlor P.G. (2021). Livestock-associated methicillin-resistant *Staphylococcus aureus* (LA-MRSA) prevalence in humans in close contact with animals and measures to reduce on-farm colonisation. Ir. Vet. J..

[46] Bourély C., Cazeau G., Jarrige N., Leblond A., Madec J.Y., Haenni M., Gay E. (2019). Antimicrobial resistance patterns of bacteria isolated from dogs with otitis. Epidemiol. Infect..

[47] Ligozzi M., Bernini C., Bonora M.G., De Fatima M., Zuliani J., Fontana R. (2002). Evaluation of the VITEK 2 system for identification and antimicrobial susceptibility testing of medically relevant gram-positive cocci. J. Clin. Microbiol..

[48] Pages Monteiro L., Von Allmen N., Friesen I., Huth K., Zambardi G. (2021). Performance of the VITEK^®^2 advanced expert system™ for the validation of antimicrobial susceptibility testing results. Eur. J. Clin. Microbiol. Infect. Dis..

[49] Bannoehr J., Ben Zakour N.L., Waller A.S., Guardabassi L., Thoday K.L., van den Broek A.H., Fitzgerald J.R. (2007). Population genetic structure of the *Staphylococcus intermedius group*: Insights into agr diversification and the emergence of methicillin-resistant strains. J. Bacteriol..

[50] Kim E., Yang S.M., Won J.E., Kim D.Y., Kim D.S., Kim H.Y. (2021). Real-Time PCR Method for the Rapid Detection and Quantification of Pathogenic *Staphylococcus* Species Based on Novel Molecular Target Genes. Foods.

[51] Bannoehr J., Franco A., Iurescia M., Battisti A., Fitzgerald J.R. (2009). Molecular diagnostic identification of *Staphylococcus pseudintermedius*. J. Clin. Microbiol..

